# The association between liking, learning and creativity in music

**DOI:** 10.1038/s41598-024-70027-z

**Published:** 2024-08-16

**Authors:** Ioanna Zioga, Peter M. C. Harrison, Marcus Pearce, Joydeep Bhattacharya, Caroline Di Bernardi Luft

**Affiliations:** 1 Donders Centre for Cognitive Neuroimaging, Donders Institute for Brain, Cognition and Behaviour, Radboud University, 6525 EN Nijmegen, The Netherlands; 2 School of Electronic Engineering and Computer Science, Queen Mary University of London, London, E1 4NS UK; 3 Faculty of Music, University of Cambridge, Cambridge, UK; 4 Department of Psychology, Goldsmiths University of London, New Cross, London, SE14 6NW UK; 5 Division of Psychology, CHMLS-Life Sciences, Brunel University London, London, UB8 3PH UK

**Keywords:** Human behaviour, Cognitive neuroscience

## Abstract

Aesthetic preference is intricately linked to learning and creativity. Previous studies have largely examined the perception of novelty in terms of pleasantness and the generation of novelty via creativity separately. The current study examines the connection between perception and generation of novelty in music; specifically, we investigated how pleasantness judgements and brain responses to musical notes of varying probability (estimated by a computational model of auditory expectation) are linked to learning and creativity. To facilitate learning de novo, 40 non-musicians were trained on an unfamiliar artificial music grammar. After learning, participants evaluated the pleasantness of the final notes of melodies, which varied in probability, while their EEG was recorded. They also composed their own musical pieces using the learned grammar which were subsequently assessed by experts. As expected, there was an inverted U-shaped relationship between liking and probability: participants were more likely to rate the notes with intermediate probabilities as pleasant. Further, intermediate probability notes elicited larger N100 and P200 at posterior and frontal sites, respectively, associated with prediction error processing. Crucially, individuals who produced less creative compositions preferred higher probability notes, whereas individuals who composed more creative pieces preferred notes with intermediate probability. Finally, evoked brain responses to note probability were relatively independent of learning and creativity, suggesting that these higher-level processes are not mediated by brain responses related to performance monitoring. Overall, our findings shed light on the relationship between perception and generation of novelty, offering new insights into aesthetic preference and its neural correlates.

## Introduction

Aesthetic preferences are complex, often balancing an inclination towards the familiar with novelty-seeking behaviour. On the one hand, individuals tend to prefer familiarity^1–4^ and high levels of surprise can elicit negative emotional responses^5^. Studies with non-human animals suggest that this preference for familiar stimuli, or conformity bias, is crucial for maintaining stable cultural traditions^6,7^. However, too much repetition can lead to boredom^8^, and humans often seek and enjoy novelty^9–12^. This pursuit of novelty is vital for development, the acquisition of new skills, and the evolution of culture and knowledge. One plausible explanation for this seeming contradiction lies in the Wundt curve of hedonic response^13^ according to which subjective preference or liking of an idea/product/piece shows an inverted U-shaped relationship with its novelty/complexity^14–16^. This means that preference and enjoyment tend to be higher for predictably intermediate stimuli: neither too familiar, which can be boring, nor too unpredictable, which might be perceived as unpleasant or incomprehensible. This inverted U-shaped pattern has been demonstrated in various domains, including music^8,17^ and visual aesthetics^13,18–20^. Nonetheless, the universality and extent of this pattern remain a topic of ongoing debate^21,22^.

This preference towards intermediate levels of novelty may benefit learning^23,24^. During learning, prediction errors (i.e. the difference between expected and actual outcomes) serve as teaching signals which trigger dopaminergic responses^25–27^. An intermediate degree of prediction error would, therefore, be an understandable teaching signal which in turn motivates learning via a manageable challenge^28^. This is demonstrated in studies showing that learning experiences are more enjoyable when they require an intermediate degree of challenge^29,30^. Hence, a preference for intermediate degrees of novelty could facilitate the learning of new information.

This link between prediction errors and learning is also evident in neuroimaging studies of music perception since the event-related-potential (ERP) components corresponding to prediction errors, such as N100 and P200, are also observed for musical stimuli^31–33^. Previous studies on music perception observed increased midfrontal negativities, like the N100, around 100 ms in response to unexpected notes^32–38^. The P200 component, peaking between 200 to 300 ms post-stimulus onset, also responds to novelty in both music and learning contexts^33,38,39^. In a previous study^33^, we observed increased N100 responses to incorrect notes and increased P200 to correct notes. These studies showed some differences regarding the topographical location of these components, often along the midline, frontal and parietal areas, which are consistent with the activity of the performance monitoring system^40–42^, crucial for updating expectations for learning.

In this context, an important question arises: how do responses to novelty in perception relate to creative abilities? Previous studies showed an association between the personality trait of openness to experience and creativity^43–46^. It could be argued that people who are more open to new experiences may enjoy higher levels of novelty since it was found that during the evaluation of creativity, individuals with higher openness placed a greater weight on novelty^47^. Creativity here is defined as the ability to produce novel, appropriate and surprising ideas^48^. Interestingly, in our previous study^33^ we observed that the P200 amplitude in response to incorrect musical notes was inversely correlated with creativity: the higher the P200 to incorrect notes, the higher the creativity of the participants' compositions. We also found that participants who learned the artificial music grammar better were more creative and their compositions contained more segments of intermediate probability, suggesting that more creative individuals may not just prefer higher levels of novelty, but are drawn to stimuli with intermediate levels of novelty, which potentially guides their creative outputs. Therefore, it is crucial to understand the relationship between perception and generation of novelty as it could elucidate the link between aesthetic enjoyment and creativity.

In the present study, we addressed this question by analysing how individual differences in perception of the pleasantness of musical notes with different probabilities (low, medium, and high) are linked to participants’ creativity in generating new melodies after learning an artificial music grammar. We addressed four specific research questions as follows: Q1: Is there an association between pleasantness judgements in music and the probability of notes, and does it follow an inverted-U shape? Q2: How do traditional ERP components, specifically N100 and P200, which are typically associated with prediction error processing, respond to notes of intermediate probability; Q3: Do individual differences in these effects correlate with the learning of the grammar and creativity of music composed by participants? Q4: Are the associations between brain responses and note probability associated with learning and creativity? We expected that: H1: Notes of intermediate probability would be rated as more pleasant than low and high probability notes, following the Wundt’s inverted-U shape; H2: Notes with intermediate probability would also result in intermediate N100 and P200 amplitudes; H3: Individuals who are better learners would show a stronger U-shaped curve regarding pleasantness judgements (due to higher sensitivity to the probabilities of the music), whereas more creative individuals would show a stronger preference for notes of intermediate probability; and finally, H4: Better learners would show stronger N100 and P200 modulation by the probability of the stimulus (e.g. higher N100 and P200 in response to notes of low probability) and that more creative individuals would show a higher N100 and P200 in response to notes of intermediate probability.

Our study uniquely addressed these questions by analysing participants’ ratings of pleasantness for musical notes following training in an unfamiliar artificial music grammar^33^. This approach enabled us to isolate probability assessments from influences like prior exposure to naturalistic music and musical training. We also evaluated creativity in musical composition using this same grammar after learning. This enabled us to test the link between learning, aesthetic judgments and creativity within the same domain; this eliminates confounding factors like verbal ability or creativity in other domains. Furthermore, by employing an artificial musical grammar and controlling note sequences, we mitigated issues related to the context in realistic music which influences music enjoyment^8^. By presenting learned melodies with the final note altered to vary in probability and ensuring these notes were new to the participants, we effectively isolated the effects of probability from repeated exposure.

## Methods

### Participants

The study involved 40 neurologically healthy adults (24 females) aged 20–32 years (mean ± s.d. age of 22.42 ± 3.04 years). The inclusion criterion required participants to be non-musicians as self-reported. To confirm this, we administered the Goldsmiths Musical Sophistication Index (Gold-MSI) questionnaire^49^, focusing on the “Musical Training” dimension to quantify participants’ level of musical training. Participants had an average musical training score of 12.09 (SD 4.60) on a scale ranging from 7 to 49 points. We removed two participants from the analysis for giving identical responses across all sessions. All participants had normal or corrected-to-normal hearing and vision and received monetary compensation at a rate of £7 per hour. All participants provided written informed consent before the start of the experiment. All methods and procedures were conducted in accordance with the ethical standards stated in the 1964 Declaration of Helsinki. The study protocol was approved by the Ethics Board at Queen Mary University of London.

### Materials

This study uses the dataset produced by an existing experiment^33^. As explained in the subsequent Procedure section, the experiment comprised 4 days. The data analyzed here are exclusively from days 3 and 4, which was neither reported nor analysed in our earlier study^33^.

### Analysis of an artificial music grammar by a model of melodic expectation

**Figure 1:**
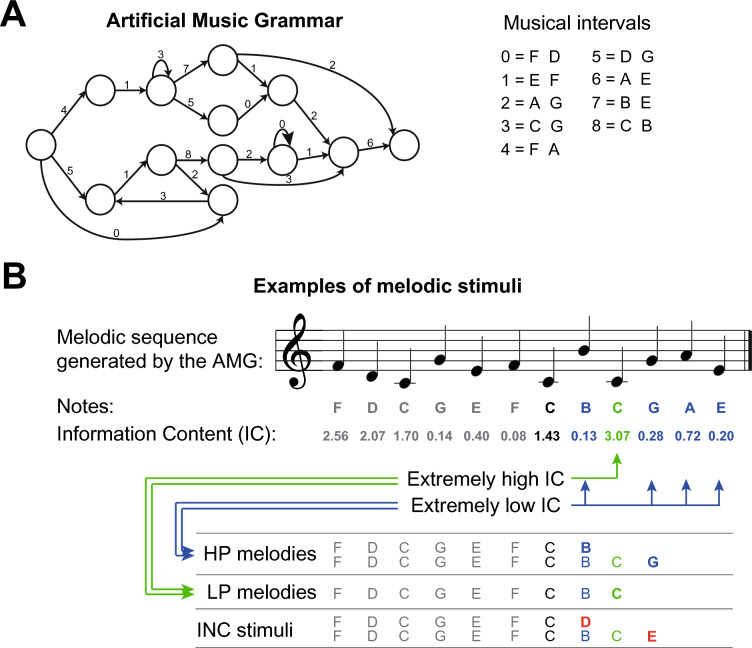
(**A**) An illustration of the artificial music grammar (AMG) developed and presented by Rohrmeier, Rebuschat, and Cross (2011). Numbers 0–8 represent the musical pairs of notes. The nodes connect the elements of the grammar (i.e. the musical intervals) with each other. Grammatical sequences start from the leftmost node and move along the pathways indicated by the arrows until the rightmost node is reached. (**B**) An example of the melodic stimuli for the test sessions. Notes of the AMG melodies with extremely high (green) and extremely low (blue) information content (IC) were identified. High-probability (HP) notes correspond to low IC, whereas low-probability (LP) notes correspond to high IC.

The stimuli comprised musical note sequences generated using an Artificial Music Grammar (AMG) developed by Rohrmeier et al.^50^ (Fig. 1A). This grammar encompasses different pairs of Western-scale diatonic notes (C4, D4, E4, F4, G4, A4, B4) to create melodic sequences. Based on the AMG, there are 18 melodic sequences ranging from 8 to 22 notes in length (with an average length of 14.56 ± 3.87 notes). Notes are defined as the 7 different pitches which are used by the AMG. It is important to note that throughout this paper, we use refer to the terms “melodic sequence” and “melody” interchangeably as each of to refer to the sequences of 8–22 notes generated using based on the rules of the artificial music grammar AMG. Twelve of those melodies, hereafter referred to as “old-grammatical”, were used during both the training and test sessions (pre-test and post-test). In contrast, six melodies, henceforth referred to as “new-grammatical”, were exclusively introduced in the final session of the experiment. This was done to evaluate participants’ judgements on melodies they had not previously heard (generalization session).

For the analysis of melodic sequences, we used the Information Dynamics of Music (IDyOM) model, an information-theoretic model of auditory expectation^51,52^. We employed a leave-one-out cross-validation approach, where predictions for each melody were generated by a model trained on all the other melodies in the corpus. This model uses different ‘viewpoints’ to capture the different cognitive representations listeners have for musical pitch. Following Pearce^51^, we selected viewpoints that minimised cross-entropy (i.e. maximised predictive performance) on the melody set. Chromatic pitch and chromatic pitch interval together (yielding a cross-entropy of 0.99) outperformed the use of chromatic pitch alone (cross-entropy of 1.01), and the combination of chromatic pitch, chromatic interval, and contour (cross-entropy of 1.04). Following Pearce (2005), IDyOM’s predictions were based on a blend of a long-term model, trained on all melodies except one and then incrementally trained on that single melody, and a short-term model, trained incrementally only on the single melody. This approach reflects the effects of both long-term learning across all stimuli and short-term learning within an individual stimulus and has been shown to effectively simulate listeners’ melodic expectations in a wide range of contexts^53–57^ including artificial grammar learning^58^. As a result, IDyOM provided probability estimates for each note in each of the 18 melodies generated by the AMG. We quantified this by calculating the information content (IC), taking the negative logarithm (base 2) of the probability estimate. Low IC values corresponded to notes with high probability (predictable notes), whereas high IC values corresponded to notes with low probability (unpredictable notes).

### Melodic stimuli

During the experiment, participants listened to melodic sequences that were interrupted at certain notes, referred to as “target notes” (Fig. 1B). In the pre-test and post-test session, participants were asked to judge whether each target note was correct or incorrect and whether it was surprising or not (according to the AMG they had learned). In both pre-test and post-test sessions, a total of 280 melodies were presented, each concluding with a target note of varying probability: 70 ended on high-probability (HP) notes, 70 ended on low-probability (LP) notes, and 70 on incorrect (INC) notes. Additionally, 70 melodies were created by shuffling the order of all notes (random melodies). At the end of the last day, participants completed a generalization session during which they needed to indicate if a target note was correct or incorrect (according to the AMG they learned) and whether it was pleasant or not (according to their taste). This session included 105 melodies: 35 melodies each for the HP, LP, and INC categories.

To create melodic sequences ending on HP and LP notes, we selected notes from the AMG melodies falling within the lowest 30% of information content (IC) (mean ± SD of 0.90 ± 0.03) for HP notes and within the highest 30% IC (mean ± SD of 0.21 ± 0.10) for LP notes. In total, this resulted in 79 notes with HP or LP, 55 belonging to the old-grammatical melodic sequences (36 notes were HP and 19 notes were LP) and 24 belonging to the new-grammatical melodic sequences (16 notes were HP and 8 notes were LP). Melodies were repeated an appropriate number of times to achieve 70 HP and 70 LP trials for the test sessions, and 35 HP and 35 LP trials for the generalization session. Specifically, we repeated 34 (random selection) of the 36 HP melodies once, whereas each of the 19 LP melodies was repeated three times (giving 57 melodies), and then 13 (randomly selected from the middle 40% of the distribution) were added. This resulted in 70 melodies for HP and 70 for LP conditions. The same procedure was followed for the new-grammatical sequences. The 16 HP melodies were repeated once and 3 (random selection from the middle 40% of the distribution) were added, while the 8 LP melodies were repeated four times and 3 (random selection from the middle 40% of the distribution) were added. This resulted in 35 melodies for HP and 35 for LP conditions.

To create melodic sequences ending on INC notes, we modified the final notes of the HP and LP melodies by replacing them with a random note—one that never appeared in that particular context in the AMG. The test sessions (pre, post) had 70 incorrect melodies each, while the generalization session included 35 incorrect melodies. The sessions included different sets of INC melodies.

The melodic stimuli were presented through speakers positioned to the left and right sides of the participants. Each note had a duration of 330 ms without intervals between notes. The notes had a piano timbre, and their fade-out duration was 100 ms. We used Psychtoolbox^59^ for the presentation of stimuli. Melodies were presented in a randomized order for each participant.

### Procedure

**Figure 2:**
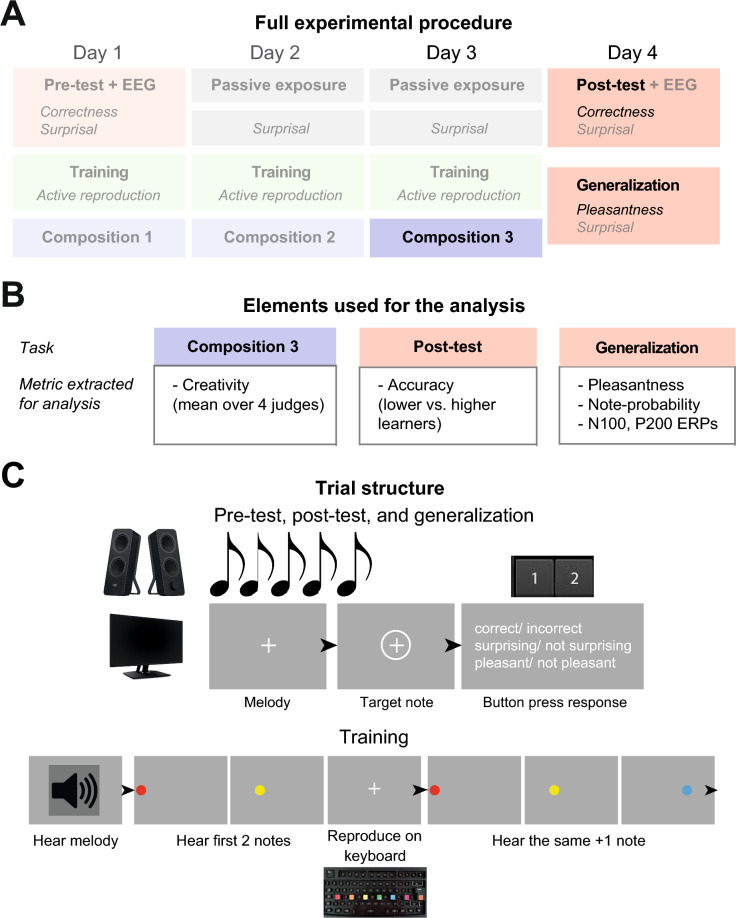
(**A**) A schematic illustration of the full experimental procedure. Opaque panels and text represent the parts used in the current paper, while transparency represents parts not used. (**Β**) The parts used in the current paper, including the respective analysis metric extracted from each of those parts. (**C**) Top: An illustration of the trial structure of the test and generalization sessions. Participants heard a melodic sequence and were asked to make judgements on target notes by pressing 1 or 2 on a computer keyboard. Bottom: An illustration of the trial structure of the three training sessions. Participants listened to a melody and needed to reproduce the notes of the melody on a sound keyboard, starting from the first two notes and adding an extra note incrementally.

Participants visited the lab on four separate days with no more than a two-day interval between the sessions (as illustrated in Fig. 2A). The musical compositions on Day 3 and the generalization sessions on Day 4 comprise the focus of the current paper.

#### Training

This session is not analyzed in the current work. During the first three days (sessions 1–3), participants underwent training on the AMG. This training involved passive listening to the melodies and reproducing them on a computer keyboard. The keys A, D, G, J, L, ‘, and ENTER were marked with coloured stickers (red, orange, yellow, green, blue, pink, brown) to represent different notes. Initially, participants listened to a melodic sequence and were presented with the first two notes, which they attempted to reproduce on the keyboard. Training progressed incrementally, with each new attempt adding an extra note to be played. If a mistake was made, the melodic segment was repeated to allow for further attempts. Each training session lasted approximately 25 min. To increase their familiarity with the AMG, on the second and third days, participants listened passively to the AMG sequences, played three times in a randomised order, while being instructed to listen attentively. Following each listening session, they were presented with melodies ending on HP or LP notes and were asked to judge whether the target note was surprising or not. Those sessions lasted around 15 min in total.

#### Pre-test and post-test

In the current paper, we analyzed only data from the post-test. The participants’ learning levels were assessed before and after the training on days 1 (pre-test) and 4 (post-test), while their electroencephalogram (EEG) was recorded (Fig. 2C). Participants were instructed to listen attentively to melodies generated by the music grammar shown in Fig. 1A. It was made clear that they would be asked to make judgements about certain target notes, regarding their correctness (correct or incorrect) and surprisal (surprising or not surprising). Specifically, participants were given the following verbal instructions: “Now you will listen to some melodies, and at certain points you will be prompted to make the aforementioned judgements about some notes. Please respond as fast and as accurately as possible”. In the pre-test, they were also told the following: “The melodies might sound odd, as they do not belong in any familiar musical genre, instead they are generated using an unfamiliar, artificial musical scale. Thus, you might feel uncertain about your judgement at the beginning, but follow your gut instinct. As the session goes by, you might start feeling more confident”. Instead, in the post-test, they were told the following instructions: “Note that you should make your judgements based on the artificial music grammar that you learned throughout the experiment, rather than based on familiar Western music”. The presentation order of the trials was randomised across participants. Each test session consisted of 280 trials and lasted approximately 40 min.

#### Generalization

Data from this session is the main focus of our paper. On day 4, during the EEG recording, participants completed a generalization session where they were presented with previously unheard sequences. They were asked to evaluate the surprisal (surprising or not surprising) and pleasantness (pleasant or not pleasant) of the notes. Participants were instructed to respond based on the rules of the artificial music grammar they were trained on. This session included a total of 105 trials and lasted 20 min.

#### Compositions

Participants’ musical composition after the last training session was used for the analysis. At the end of each training session, participants were asked to create one musical composition. They were advised to compose something based on what they learned and were prompted to be as creative as possible. They were given 3 min to prepare their composition while playing on the keyboard and using pen and paper. They were then given 20 s to perform their composition on the keyboard. The performances were recorded through MATLAB^®^. The creativity of those compositions was subsequently evaluated by expert musicians.

### EEG recording and preprocessing

EEG signals were recorded using 64 Ag–AgCl electrodes attached to the EGI geodesic sensor net system (HydroCel GSN 64 1.0; EGI System 200; Electrical Geodesic Inc., OR, USA; https://www.egi.com/). The data were amplified by an EGI Amp 300 and sampled at 500 Hz. We used the MATLAB Toolbox EEGLAB^60^ for data preprocessing and FieldTrip^61^ for data analysis. Signals were recorded with an online reference at the right mastoid and re-referenced to the average of the left and right mastoids. Continuous data were high-pass filtered at 0.5 Hz and then epoched from − 100 to 500 ms around the onset of target notes. Channels with poor signal quality, as assessed by visual inspection and by studying the topographical maps of their power spectra, were interpolated using neighbouring electrodes. Epochs containing artifacts such as movement, muscle activity and saccades were removed after visual inspection. Epochs with eye-blink artefacts were corrected using independent component analysis. Finally, epochs were low-pass filtered at 15 Hz and baseline corrected from − 100 to 0 ms before the target note onset. Participants with fewer than 10 useable trials in any of these three conditions were excluded, resulting in a final sample of 32 participants.

### Creativity evaluation of participants’ compositions

After the end of the data collection, four expert classically-trained musicians were recruited to evaluate the creativity of the musical compositions. All musicians had a minimum of 10 years of formal musical training in a conservatory. Before the evaluations, they received training on the AMG to familiarize themselves with the music. Judges were given a verbal description of the creativity concept, i.e. that creativity is defined as the combination of both novel and correct (based on the AMG) elements, and were instructed to follow the definition as well as their gut instinct for completing the evaluation. Importantly, judges were instructed to provide ratings considering the constraints of the AMG. They used a scale ranging from 1 (not at all creative) to 5 (extremely creative) for their evaluations. There was a reasonable agreement between the four raters, as indicated by the interclass correlation coefficient, ICC = 0.56 (confidence interval [0.47–0.65]). Z-scores were calculated separately for each judge individually and then averaged across all four judges, this was done to ensure they were normalized across their base rates.

### Data analysis

To address Q1 (Is there an association between pleasantness judgements in music and the probability of notes, and does it follow an inverted-U shape?) we split the notes according to their probability using two different methods (to ensure our results were not an artefact of our binning method): (1) The notes were divided into 9 bins according to their probability, using a sliding window of 20% for each decile (0–20%, 10–30%, 20–40%, etc.). We then calculated the proportion of notes identified as pleasant (i.e. the number of pleasant instances in the i-th bin divided by the number of trials in the i-th bin) and entered them in a one-way repeated-measures ANOVA, with note probability as the independent variable with 9 levels (bins). (2) For the second method, we split the notes into three different probability levels (low: 0 to 0.33, medium: 0.33 to 0.66, high: 0.66 to 1). We then calculated the percentage of notes rated as pleasant and entered this into a one-way repeated-measures ANOVA with three levels of probability (low, medium, and high). We looked at both linear and quadratic effects since we expected the association to follow Wundt's inverted-U shape.

To address Q2 (How do traditional ERP components, specifically N100 and P200, which are typically associated with prediction error processing, respond to notes of intermediate probability?), we calculated the peak amplitudes of two main ERP components: N100 (peak negative amplitude between 50 and 150 ms after the note onset) and P200 (peak positive amplitude between 100 and 250 ms after the note onset) over two different regions (fronto-central—FCz and centro-posterior—PCz). We focused on the N100 and P200 at midline electrodes since these components are sensitive to prediction errors^41,62^ and have been observed in studies of music perception^31,33,36,38^. We chose FCz and PCz specifically because they are distant enough from each other to be differentially sensitive to frontal and mid-parietal influences without being too affected by prefrontal and occipital sources, respectively. The time windows used to identify the peaks and the regions were defined by visually inspecting the average ERP waveforms observed in these two regions (Fig. 4A). We entered the peak amplitude into a repeated-measures ANOVA with note probability (low, medium, high) as the independent variable. Separate analyses were carried out for the N100 and P200 peak amplitudes at each electrode region (FCz and PCz).

To address Q3 (Do individual differences in these effects correlate with the learning of the grammar and creativity of music composed by participants?), we split the learning groups into two (lower vs. higher learners) based on a median split of their accuracy in the post-test. Learning was assessed by calculating the accuracy of a participant’s judgments of whether a note was correct or incorrect following learning of the artificial musical grammar. Creativity was measured as the average creativity rating of their compositions given by four musical experts in Day 3 relative to the musical grammar learned (see “Methods”). We split the participants into two creativity groups based on a median split of their creativity, resulting in two equal groups (lower vs. higher creativity). To investigate the individual differences at the behavioural level, we entered the average ratings of the pleasantness of the unseen notes (at post-test) in a 2 (learning: lower vs. higher) × 2 (creativity: lower vs. higher) × 3 (note probability: low, medium, high) mixed design ANCOVA. We entered the pleasantness ratings (liking) for these compositions as a control variable to avoid confounding creativity with how pleasant the compositions sounded to the raters since these two variables (pleasantness and creativity of their compositions) were highly correlated (r = 0.907, p < 0.001). We looked at linear and quadratic effects due to our hypothesis regarding the Wundt inverted-U shape association. To investigate the individual differences at the behavioural level, we conducted the same ANCOVA but using the N100 and P200 as dependent variables (one analysis per dependent variable).

To address Q4 (Are the associations between brain responses and note probability associated with learning and creativity?), we applied the same statistical test as in Q3 but with the ERP components of interest. Therefore, we entered the centro-posterior N100 and the fronto-central P200 as dependent variables in a 2 (learning: lower vs. higher) × 2 (creativity: lower vs. higher) × 3 (note probability: low, medium, high) mixed design ANCOVA, with pleasantness as a covariate.

## Results

### Liking notes of different probability

**Figure 3:**
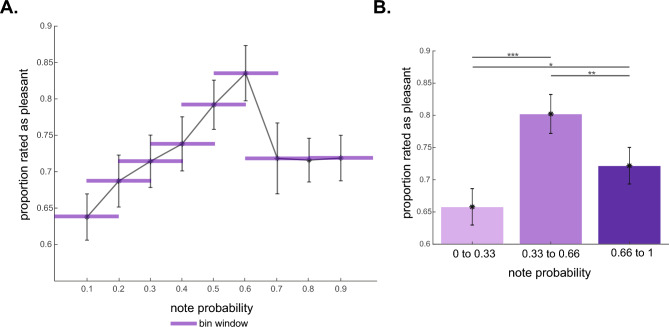
Pleasantness ratings for notes with varying probability. (**A**) Proportion of notes rated as pleasant within each probability bin with a 50% overlap. The proportion was calculated per bin. The purple shades represent the bin width. (**B**) Proportion of notes rated as pleasant for each non-overlapping probability bin. Error bars represent 1 ± SEM *p < 0.05; **p < 0.01; ***p < 0.001.

Figure 3A shows the relationship between the proportion of pleasant notes and note probability, suggesting that participants tend to rate more probable notes as more pleasant but also an inverted-U shaped effect such that pleasure falls for very high probabilities. We conducted a one-way repeated-measures ANOVA on the proportion of pleasant notes, with note probability as the independent variable with 9 levels (bins). The results show a significant effect of probability on pleasantness ratings (*F*_(8,312)_ = 6.527, *p* < 0.001, *partial η*^2^ = 0.143). The within-subject contrasts revealed a strong quadratic effect (*F*_(1,39)_ = 21.967, *p* < 0.001, *partial η*^2^ = 0.360), which was more pronounced than the significant linear contrast (*F*_(1,39)_ = 6.660, *p* = 0.014, *partial η*^2^ = 0.146). Pairwise contrasts between each probability bin revealed that the highest pleasantness ratings were for notes with intermediate probabilities, particularly in the range of 0.4 and 0.6 (*p* < 0.05). As shown in Fig. 3A, notes with the lowest probabilities (0–0.2) were rated as the least pleasant, while the most pleasant notes fell within the 0.4–0.6 probability range.

To verify that the observed effect was not simply due to our binning method, we conducted a similar analysis (Fig. 3B) using the three broad probability ranges (low: 0–0.33, medium: 0.33–0.66, high: 0.66–1). The percentage of notes rated as pleasant was entered into a one-way repeated-measures ANOVA with three levels of probability (low, medium, and high). The results show a significant main effect of probability (*F*_(2,78)_ = 18.749, *p* < 0.001, *partial η*^2^ = 0.325). The within-subjects contrasts showed that the quadratic effect (*F*_(1,39)_ = 24.394, *p* < 0.001, *partial η*^2^ = 0.385) was stronger than the linear effect (*F*_(1,39)_ = 10.892, *p* = 0.002, *partial η*^2^ = 0.218). Follow-up pairwise contrasts revealed that notes with intermediate probability (0.33–0.66) were considered significantly more pleasant than both the less probable notes (*t*_(39)_ = 5.395, *p* < 0.001, *Cohen’s d* = 0.853) and the more probable notes (*t*_(39)_ = 3.319, *p* = 0.002, *Cohen’s d* = 0.525). Finally, the most probable notes (greater than 0.66 probability) were rated as significantly more pleasant than the least probable ones (*t*_(39)_ = 3.30, *p* = 0.002, *Cohen’s d* = 0.512).

The incorrect notes were excluded from the previous analyses because they not only have a much lower probability (mean ± S.D. probability = 0.022 ± 0.002) compared to the low probability correct notes (0.161 ± 0.009) but they are also ungrammatical; the latter issue could bias the results and spuriously exaggerate the effects. To address this, we conducted another one-way repeated measures ANOVA with four levels of probability (very low and incorrect, low, medium, high, all correct) to compare preferences between incorrect and varying levels of correct note probabilities. As expected, we observed a significant main effect of probability (*F*_(3,117)_ = 36.241, *p* < 0.001, *partial η*^2^ = 0.482). With the inclusion of incorrect notes as the lowest probability level, the linearity of the effect increased (*F*_(1,39)_ = 50.386, *p* < 0.001, *partial η*^2^ = 0.564) in the within-subjects contrasts, while the quadratic effect remained strong (*F*_(1,39)_ = 34.696, *p* < 0.001, *partial η*^2^ = 0.471). Participants found the incorrect notes less pleasant than those of medium probability (*t*_(39)_ = 7.660, *p* < 0.001, *Cohen’s d* = 1.21), as well as compared to the high (*t*_(39)_ = 6.419, *p* < 0.001, *Cohen’s d* = 1.01) and low probability notes (*t*_(39)_ = 4.943, *p* < 0.001, *Cohen’s d* = 0.995).

### ERPs in response to low, medium, and high probability notes

We investigated the differences in brain response to each note according to probability, specifically looking at whether variations between low, medium, and high probability notes would manifest in event-related potentials (ERPs). First, we investigated the main differences in the P200 and N100 components at the fronto-central midline (FCz) and centro-posterior midline (PCz) between notes with medium (0.33–0.66), high (> 0.66), and low (< 0.33) probability.

**Figure 4:**
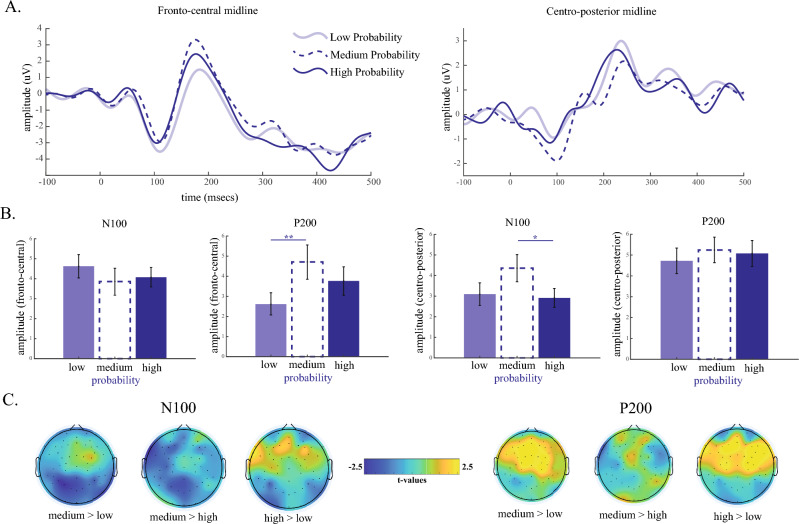
ERPs in response to notes of low, medium and high-probability (probability ranges from 0–0.33, 0.33–0.66, 0.66–1). (**A**) ERP waveforms at the: fronto-central midline electrode (left-hand side) and at the centro-posterior midline (right-hand side) for notes of low, medium and high-probability. (**B**) Mean N100 and P200 peak amplitude for each non-overlapping probability bin. Error bars represent 1 ± S.E.M. *p < 0.05; **p < 0.01; ***p < 0.001. (**C**) Topographical distribution of the difference in N100 (left-hand side) and P200 (right-hand side) for the contrasts: medium vs. low, medium vs. high and high vs. low probability. The difference is quantified as the t-values at each electrode location.

For the N100 component at the frontal midline region (Fig. 4B, left-hand side), we observed no significant main effect of note probability on peak amplitude (*F*(2,62) = 0.597, *p* = 0.553, *partial η*^2^ = 0.019), neither a linear nor quadratic effect (*p* > 0.2). However, at the centro-posterior midline region, we found a significant main effect of probability (*F*(2,62) = 3.540, *p* = 0.035, *partial η*^2^ = 0.103). The quadratic effect of probability was significant (*F*(1,31) = 5.182, *p* = 0.030, *partial η*^2^ = 0.143), but the linear effect was not (*F*(1,31) = 0.510, *p* = 0.480, *partial η*^2^ = 0.016). Pairwise contrasts showed that the N100 amplitude was significantly higher for notes with medium compared to those with high probability (*t*(31) = 2.360, *p* = 0.025, *Cohen’s D* = 0.417). A marginal difference was observed between medium and low probability notes (*t*(31) = 1.827, *p* = 0.077, *Cohen’s D* = 0.323), but no significant difference was found between low and high probability notes (*t*(31) = 0.714, *p* = 0.480, *Cohen’s D* = 0.126).

For the P200 component at the fronto-central midlines, we observed a significant main effect of probability (*F*(2,62) = 4.391, *p* = 0.016, *partial η*^2^ = 0.124). As with the behavioural data, within-subjects contrasts showed a significant quadratic effect (*F*(1,31) = 4.813, *p* = 0.036, *partial η*^2^ = 0.134), whilst the linear effect was non-significant (*F*(1,31) = 3.720, *p* = 0.063, *partial η*^2^ = 0.107). Follow-up pairwise contrasts showed a significant difference in the P200 peak amplitude for medium versus low probability notes (*t*(31) = 2.791, *p* = 0.009, *Cohen’s D* = 0.493). However, no significant difference was found between medium and high probability notes (*t*(31) = 1.195, *p* = 0.241, *Cohen’s D* = 0.211) and only a marginal difference between low and high probability notes (*t*(31) = 1.941, *p* = 0.061, *Cohen’s D* = 0.343). At the centro-parietal midline region, we observed no effect of probability on the P200 (*F*(2,62) = 0.098, *p* = 0.908, *partial η*^*2*^ = 0.003), with both quadratic and linear effects being non-significant (*p* > 0.5 for both).

### Association between learning, creativity, and preference for novelty

**Figure 5:**
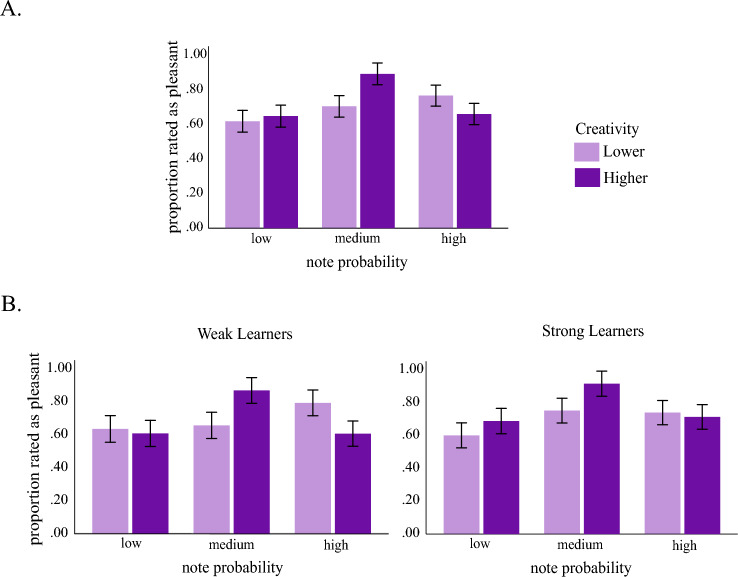
Associations between pleasantness judgments, note probability, creativity and learning. (**A**) Proportion of notes with low, medium and high probability rated as pleasant in groups with lower (light purple) and higher (dark purple) creativity. (**B**) Same as in (**A**), but for weak and strong learners separately. Error bars represent 1 ± S.E.M.

Finally, we aimed to understand how learning and creativity are associated with preference for different levels of novelty. The results confirmed a strong effect of probability on liking (*F*(2,66) = 24.597, *p* < 0.001, *partial η*^2^ = 0.427), and a significant interaction between note probability and creativity (F(2,66) = 6.587, *p* = 0.002, *partial η*^2^ = 0.166). This interaction showed a strong quadratic effect (*F*(1,33) = 9.754, *p* = 0.004, *partial η*^2^ = 0.228). This interaction seems to be due to the more creative group showing a stronger liking for notes of intermediate probability compared to the notes with lower and higher probability (Fig. 5A). There was no interaction between note probability and learning (*F*(2,66) = 0.598, *p* = 0.553, *partial η*^2^ = 0.018), nor a three-way interaction (*F*(2,66) = 2.584, *p* = 0.083, *partial η*^2^ = 0.073). However, the within-subjects effects showed a significant quadratic three-way interaction (*F*(1,33) = 4.663, *p* = 0.038, *partial η*^2^ = 0.124), which might be attributed to a slightly lower quadratic effect of probability within participants with lower creativity who were also weak learners (Fig. 5B).

We then tested whether the centro-posterior N100 and fronto-central P200, which were found to be associated with the probability of the notes, were associated with learning and creativity by entering these measures (separately) as dependent variables in a 2 (learning: lower vs. higher) × 2 (creativity: lower vs. higher) × 3 (note probability: low, medium, high) mixed design ANCOVA, with pleasantness as a covariate. As expected for the N100, we observed a significant effect of probability (F(2,50) = 3.414, *p* = 0.041, *partial η*^2^ = 0.120), with a significant quadratic effect (F(1,25) = 4.929, *p* = 0.036, *partial η*^2^ = 0.165), but note probability did not interact with creativity (F(2,50) = 0.191, *p* = 0.826, *partial η*^2^ = 0.008) nor learning (F(2,50) = 0.616, *p* = 0.544, *partial η*^2^ = 0.024). We observed the same effects for the P200 (fronto-central) with only a significant main effect of note probability (F(2,50) = 3.475, *p* = 0.038, *partial η*^2^ = 0.118), but no interaction with creativity (F(2,50) = 1.140, *p* = 0.328, *partial η*^2^ = 0.042) or learning (F(2,50) = 2.186, *p* = 0.123, *partial η*^2^ = 0.078).

To ensure that our results were not due to potential motor preparation differences between conditions (as the participants were asked to respond as fast as possible after the tone), we analysed the participants response times to notes of low, medium, and high probability. If motor contamination is a confound, we expected to find a difference in response times between the conditions. On average, participants took approximately 1 s to respond (mean = 0.99 s, SD = 0.59). We then entered the response times in a repeated measures ANOVA with three levels: low, medium, and high probability using the response times as the dependent variable. We observed no significant difference between the conditions (*F*_(2,78)_ = 0.717, *p* = 0.493, *partial η*^2^ = 0.018).

## Discussion

In this study, we investigated the relationship between aesthetic enjoyment and creativity focusing on novelty perception and generation. After receiving training on melodies generated by an artificial music grammar, participants evaluated the pleasantness of short melodic excerpts ending with notes of varying probability while their EEG responses were recorded. They also composed their own musical compositions, whose creativity was later assessed by experts. Our key findings include: (1) in response to our research question 1 (RQ1), we found an inverted U-shaped relationship between note-pleasantness and note-probability; (2) in response to RQ2, we observed that intermediate probability notes were associated with brain responses traditionally linked to prediction error, namely higher N100 and P200 at midline posterior and frontal sites, respectively; (3) regarding RQ3, more creative individuals preferred notes with intermediate levels of probability, whereas less creative individuals preferred higher probability notes; (4) and finally in response to RQ4, we observed no interaction between note-probability, creativity or learning on the N100 and P200, suggesting that those event related responses are not the main brain mechanisms behind this association.

As hypothesized, participants found notes of intermediate probability as the most pleasant, while low- and high-probability notes were judged as less pleasant. This inverted U-shaped relationship is in line with previous research on liking and complexity, where stimuli with intermediate complexity were liked more^8,63^. This relationship was proposed in the 1960s by Daniel Berlyne (Berlyne, 1960), who argued that aesthetic preference is influenced by the balance between familiarity/novelty and complexity (collative variables) as people tend to show a preference for intermediate levels of these quantities. The inverted U-shaped relationship between pleasantness and complexity is further explained by the predictive coding account of learning, proposing that the brain uses Bayesian inference to constantly generate and update predictions about both external input and internal states, based on generative internal models^65^: prediction errors reflect mismatches between prediction and actual input, thus forcing refinements to these internal models. These prediction errors are then used as guiding signals for learning where intermediate degrees of complexity offer the greatest opportunities for learning^66^.

Regarding aesthetic preference and pleasantness in music, prominent theories emphasize the significance of prediction violation and confirmation^67–72^. In music, the predictive processes are thought to generate reward^8,73,74^ through actively generating and updating predictions^75^. Recently, Kathios et al.^76^ found evidence for the predictive coding account showing an inverted U-shaped relationship between liking and exposure (number of presentations), as well as a higher liking for melodies with fewer prediction errors. Similar to our study, the authors used an unfamiliar music scale, the Bohlen-Pierce scale, which is different from any established musical culture. Importantly, they also demonstrated these effects in both American and Chinese participants, underlining the potential cultural independence of these findings.

In the present experiment, training non-musicians on an artificial music grammar allowed us to study the effects of learning and music expectation on preference, avoiding biases accumulated from prior musical exposure. Music prediction errors have been associated with increased brain activity in auditory and reward-related areas, including amygdala, hippocampus, and ventral striatum^8,63^. Further, increased functional connectivity between auditory and reward networks has been linked to repeated exposure and liking^76^. Therefore, the reported inverted U-shaped relationship between pleasantness and note probability might reflect a preference for intermediate arousal, evoked by notes that are neither completely unexpected nor entirely predictable. According to the predictive coding framework, predictive processes generate reward through learning which motivates updating the internal model for improved future prediction. Whereas high probability notes are not informative, simply confirming an existing prediction, low probability notes are informative but also might signal an incorrect underlying predictive model of the sensory environment. Notes with intermediate probability are informative enough to drive learning without being so surprising as to question the underlying predictive model.

Interestingly, intermediate probability notes were associated with brain responses traditionally linked to prediction errors: in particular, the N100 and P200 amplitudes at posterior and frontal sites, respectively, were more pronounced for these notes. The fronto-central N100, in particular, has been associated with violations of melodic expectation^33,36,38,77–79^. Unexpected notes have also been found to elicit electrophysiological indicators of surprise processing, such as the mismatch negativity^80,81^, the P300 component^81,82^, the P200 component at frontotemporal sites^78^, and a late negativity around 400 ms^83,84^. Furthermore, neural activity over frontotemporal sensors has been found to track melodic surprise around 200 ms and 300–500 ms after note onset^85^. These responses have been observed for various kinds of auditory regularity violation, including out-of-key notes embedded in chords^86^, unexpected chords^87^, and unexpected notes^78,83^. These findings support the theory of expectation suppression, where expected stimuli elicit weaker neural responses than unexpected stimuli through by top-down predictive processes^88,89^.

We postulate that the observed enhancement of N100 and P200 amplitudes for intermediate probability notes could reflect the efficiency of predictive processes. These components responded more strongly to intermediate probability notes compared to high probability notes. This heightened neural responses to intermediate probability notes may be due to their role in refining the internal model and optimizing predictions, in contrast to highly expected or unexpected notes. However, since the spatial location of the N100 response we observed at the centro-posterior sites differs from prior studies reporting it at more frontal sites, future research will be needed to elucidate the distinct functional relevance of the N100 component based on its spatial distribution. Importantly, visual inspection of our topographical maps show that the right frontal area seem to scale linearly with the prediction error (high > medium > low), so it could be that whilst some brain areas have a role refining and optimizing predictions, others might be more sensitive to the degree of prediction error. It would also be important to independently replicate this finding since we took a more exploratory approach by testing the effects on both fronto-central and centro-posterior locations.

Our study also revealed that more creative individuals preferred notes with intermediate levels of probability, whereas less creative individuals preferred higher probability notes. This is partially in line with previous research demonstrating a greater tolerance for unexpected or incorrect stimuli in musicians compared to non-musicians^33,90^. In our earlier study^33^, we found an enhanced P200 to incorrect notes compared to correct ones. It was also demonstrated^90^ that jazz improvisers indicate a higher preference for unexpected chord progressions compared to classical musicians and non-musicians, who preferred expected chords. This supports the idea that experts prefer more complex structures than those with less domain-specific knowledge although that has not been always replicated^91^. This may be because individuals with higher creativity have a higher tolerance or even preference for surprising events, whereas less creative individuals react strongly to incorrect notes. As creativity involves thinking outside the box and rule-breaking, a higher tolerance for the unexpected might facilitate creative thinking by enabling access to alternative, uncommon possibilities. Nonetheless, in our study, we observed that more creative individuals preferred intermediate levels of probability rather than higher levels of novelty, which is aligned with Berlyne’s^64^ inverted-U curve of aesthetic preference. Perhaps rather than breaking rules, creative thinking in music could require an ability to introduce sufficient novelty to avoid boredom, generate interest, and maximise pleasure, but not so much as to overload a listener’s predictive model.

Finally, evoked brain responses to note probability seemed relatively independent of individual differences in creativity and learning of the new musical grammar. This is surprising, considering a previous study^90^ demonstrating that jazz musicians showed stronger evoked responses to unexpected chord progression (higher ERAN and P3b) which correlated with creativity in validated creativity tasks. We suggest several potential explanations for this. One possibility is that the absence of effects of learning and creativity on the evoked brain responses to probability might be due to the brain measures used. The N100 and P200 are evoked responses to the note played, which we found to be related to liking/preference. However, higher-level cognitive processes such as creativity and learning might not be captured by evoked responses that are time- and phase-locked to stimuli. Higher-level cognitive processes associated with manipulating learned probabilities might be more relevant for explaining learning and creativity. For instance, musical improvisation has been linked to the deactivation of a distributed brain network of prefrontal regions^92^, while music composition involves increased connectivity between the anterior cingulate cortex and the default mode network^93^. Another possibility is that learning and creativity affect brain responses to properties of music other than probability. Alternatively, the processes involved in perceiving probability may not be directly linked to generating novelty; the brain responses involved in encoding novelty could be relatively independent of those involved in generating it. A third explanation is that our non-musician sample was not equally proficient in perceiving vs. generating novelty, given their limited knowledge of the artificial music grammar, which would not be the case with expert musicians. Future research with carefully designed experiments involving expert musicians will be needed to isolate the most likely explanation.

Our study is not without limitations. First, the melodic sequences were generated from an artificial music grammar, which helped eliminate the effect of pre-existing biases (ensuring zero stylistic familiarity) and focused solely on melody. However, it is important to note that other musical dimensions like rhythm, dynamics, and instrumentation, which we did not include, also play a crucial role in influencing musical pleasure^8,94^. Second, future studies are needed to investigate individual differences in the experience of musical reward, particularly in the context of using music for emotional evocation or mood regulation, and examine their effects on brain responses to music expectations. Third, the binary nature of the reported pleasantness judgment (pleasant or not pleasant) was somewhat restrictive; a more nuanced, graded scale might have yielded richer insights. Lastly, although we have speculated about a predictive coding account for the perception of pleasantness of music, future studies are needed to validate this hypothesis across different modalities and with more complex stimuli.

## Conclusion

Overall, our study offers novel contributions to the understanding of musical pleasure and its neural correlates by employing an artificial music scale to eliminate pre-existing biases. We showed an inverted U-shaped relationship between the perceived pleasantness of a note and its probability, alongside insights into the neural mechanisms associated with these processes. Assuming a link between pleasantness and reward, our results support a predictive coding framework where the reward is derived from the active generation and updating of predictions linked to note probabilities. Furthermore, we found that the N100 and P200 components were associated with intermediate note probabilities, thereby suggesting that aspects of musical pleasure emerge even at the early stages of sensory processing. Finally, our study indicates that more creative participants showed a preference for notes with intermediate probability, partially in line with previous evidence for an expanded palette of options and possibilities in individuals with higher creativity, but offering support to an inverted U-curve for aesthetics enjoyment in more creative individuals. Ultimately, our findings support the idea that individual preference for intermediate probabilities might be also important for producing creative outputs.

### Supplementary Information

Supplementary Figure S1.

## Data Availability

The data underpinning this publication is available at https://osf.io/568jy/.

## References

[1] Burgess, T. D. II. & Sales, S. M. Attitudinal effects of “mere exposure”: A reevaluation. *J. Exp. Soc. Psychol.* **7**, 461–472 (1971). 10.1016/0022-1031(71)90078-3

[2] Hunter, P. G. & Schellenberg, E. G. Interactive effects of personality and frequency of exposure on liking for music. *Personal. Individ. Differ.* **50**, 175–179 (2011). 10.1016/j.paid.2010.09.021

[3] Tan, S.-L., Spackman, M. P. & Peaslee, C. L. The effects of repeated exposure on liking and judgments of musical unity of intact and patchwork compositions. *Music. Percept.* **23**, 407–421 (2006). 10.1525/mp.2006.23.5.407

[4] Zajonc, R. B. Attitudinal effects of mere exposure. *J. Pers. Soc. Psychol.* **9**, 1 (1968).5667435 10.1037/h0025848

[5] Egermann, H., Pearce, M. T., Wiggins, G. A. & McAdams, S. Probabilistic models of expectation violation predict psychophysiological emotional responses to live concert music. *Cogn. Affect. Behav. Neurosci.* **13**, 533–553 (2013). 23605956 10.3758/s13415-013-0161-y

[6] Lachlan, R. F., Ratmann, O. & Nowicki, S. Cultural conformity generates extremely stable traditions in bird song. *Nat. Commun.* **9**, 2417 (2018). 29925831 10.1038/s41467-018-04728-1 PMC6010409

[7] Williams, H. & Lachlan, R. F. Evidence for cumulative cultural evolution in bird song. *Philos. Trans. R. Soc. B* **377**, 20200322 (2022). 10.1098/rstb.2020.0322 PMC8666912 34894731

[8] Gold, B. P., Pearce, M. T., Mas-Herrero, E., Dagher, A. & Zatorre, R. J. Predictability and uncertainty in the pleasure of music: A reward for learning?. *J. Neurosci.* **39**, 9397–9409 (2019). 31636112 10.1523/JNEUROSCI.0428-19.2019 PMC6867811

[9] Chmiel, A. & Schubert, E. Unusualness as a predictor of music preference. *Musicae Scientiae* **23**, 426–441 (2019). 10.1177/1029864917752545

[10] Costa, V. D., Tran, V. L., Turchi, J. & Averbeck, B. B. Dopamine modulates novelty seeking behavior during decision making. *Behav. Neurosci.* **128**, 556 (2014). 24911320 10.1037/a0037128 PMC5861725

[11] Song, J., Kwak, Y. & Kim, C.-Y. Familiarity and novelty in aesthetic preference: The effects of the properties of the artwork and the beholder. *Front. Psychol.* **12**, 694927 (2021). 34367021 10.3389/fpsyg.2021.694927 PMC8345014

[12] Wang, Y. *et al.* Novelty seeking is related to individual risk preference and brain activation associated with risk prediction during decision making. *Sci. Rep.* **5**, 10534 (2015). 26065910 10.1038/srep10534 PMC4464254

[13] Berlyne, D. E. *Aesthetics and Psychobiology*, vol. 336 (Appleton-Century-Crofts, 1971).

[14] Margulis, E. H. & Beatty, A. P. Musical style, psychoaesthetics, and prospects for entropy as an analytic tool. *Comput. Music J.* **32**, 64–78 (2008). 10.1162/comj.2008.32.4.64

[15] Wiggins, G. A., Tyack, P., Scharff, C. & Rohrmeier, M. The evolutionary roots of creativity: Mechanisms and motivations. *Philos. Transa. R. Soc. B Biol. Sci.* **370**, 20140099 (2015). 10.1098/rstb.2014.0099 PMC4321140 25646522

[16] Chmiel, A. & Schubert, E. Back to the inverted-U for music preference: A review of the literature. *Psychol. Music* **45**, 886–909 (2017). 10.1177/0305735617697507

[17] Delplanque, J., De Loof, E., Janssens, C. & Verguts, T. The sound of beauty: How complexity determines aesthetic preference. *Acta Psychol.* **192**, 146–152 (2019). 10.1016/j.actpsy.2018.11.011 30504052

[18] Farley, F. H. & Weinstock, C. A. Experimental aesthetics: Children’s complexity preference in original art and photoreproductions. *Bull. Psychonomic Soc.* **15**, 194–196 (1980). 10.3758/BF03334506

[19] Imamoglu, Ç. Complexity, liking and familiarity: Architecture and non-architecture Turkish students’assessments of traditional and modern house facades. *J. Environ. Psychol.* **20**, 5–16 (2000). 10.1006/jevp.1999.0155

[20] Tinio, P. P. L. & Leder, H. Just how stable are stable aesthetic features? Symmetry, complexity, and the jaws of massive familiarization. *Acta Psychol.* **130**, 241–250 (2009). 10.1016/j.actpsy.2009.01.001 19217589

[21] Güçlütürk, Y., Jacobs, R. H. & van Lier, R. Liking versus complexity: Decomposing the inverted U-curve. *Front. Hum. Neurosci.* **10**, 112 (2016). 27047359 10.3389/fnhum.2016.00112 PMC4796011

[22] Nadal, M., Munar, E., Marty, G. & Cela-Conde, C. J. Visual complexity and beauty appreciation: Explaining the divergence of results. *Empir. Stud. Arts* **28**, 173–191 (2010). 10.2190/EM.28.2.d

[23] Matthews, T. E., Stupacher, J. & Vuust, P. The pleasurable urge to move to music through the lens of learning progress. *J. Cogn.* **6**, (2023). 10.5334/joc.320 PMC10503533 37720891

[24] Ortiz-Tudela, J. *et al.* Not what u expect: Effects of prediction errors on item memory. *J. Exp. Psychol. Gen.* **152**, 2160–2176 (2023). 36996155 10.1037/xge0001367

[25] Abler, B., Walter, H., Erk, S., Kammerer, H. & Spitzer, M. Prediction error as a linear function of reward probability is coded in human nucleus accumbens. *Neuroimage* **31**, 790–795 (2006). 16487726 10.1016/j.neuroimage.2006.01.001

[26] Schultz, W. Dopamine reward prediction error coding. In *Dialogues in Clinical Neuroscience* (2022). 10.31887/DCNS.2016.18.1/wschultz PMC4826767 27069377

[27] Steinberg, E. E. *et al.* A causal link between prediction errors, dopamine neurons and learning. *Nat. Neurosci.* **16**, 966–973 (2013). 23708143 10.1038/nn.3413 PMC3705924

[28] Bromberg-Martin, E. S., Matsumoto, M. & Hikosaka, O. Dopamine in motivational control: Rewarding, aversive, and alerting. *Neuron* **68**, 815–834 (2010). 21144997 10.1016/j.neuron.2010.11.022 PMC3032992

[29] Abuhamdeh, S. & Csikszentmihalyi, M. The importance of challenge for the enjoyment of intrinsically motivated, goal-directed activities. *Personal. Soc. Psychol. Bull.* **38**, 317–330 (2012). 10.1177/0146167211427147 22067510

[30] Abuhamdeh, S. & Csikszentmihalyi, M. Attentional involvement and intrinsic motivation. *Motiv. Emot.* **36**, 257–267 (2012). 10.1007/s11031-011-9252-7

[31] Abla, D., Katahira, K. & Okanoya, K. On-line assessment of statistical learning by event-related potentials. *J. Cogn. Neurosci.* **20**, 952–964 (2008). 18211232 10.1162/jocn.2008.20058

[32] Loui, P., Wu, E. H., Wessel, D. L. & Knight, R. T. A generalized mechanism for perception of pitch patterns. *J. Neurosci.* **29**, 454–459 (2009). 19144845 10.1523/JNEUROSCI.4503-08.2009 PMC2779050

[33] Zioga, I., Harrison, P. M. C., Pearce, M. T., Bhattacharya, J. & Di Bernardi Luft, C. From learning to creativity: Identifying the behavioural and neural correlates of learning to predict human judgements of musical creativity. *NeuroImage* **206**, 116311 (2020). 31669411 10.1016/j.neuroimage.2019.116311

[34] Carrus, E., Pearce, M. T. & Bhattacharya, J. Melodic pitch expectation interacts with neural responses to syntactic but not semantic violations. *Cortex* **49**, 2186–2200 (2013). 23141867 10.1016/j.cortex.2012.08.024

[35] Koelsch, S., Busch, T., Jentschke, S. & Rohrmeier, M. Under the hood of statistical learning: A statistical MMN reflects the magnitude of transitional probabilities in auditory sequences. *Sci. Rep.* **6**, 19741 (2016). 26830652 10.1038/srep19741 PMC4735647

[36] Koelsch, S. & Jentschke, S. Differences in electric brain responses to melodies and chords. *J. Cogn. Neurosci.* **22**, 2251–2262 (2010). 19702466 10.1162/jocn.2009.21338

[37] Omigie, D., Pearce, M. T. & Stewart, L. Tracking of pitch probabilities in congenital amusia. *Neuropsychologia* **50**, 1483–1493 (2012). 22414591 10.1016/j.neuropsychologia.2012.02.034

[38] Zioga, I., Harrison, P. M. C., Pearce, M. T., Bhattacharya, J. & Luft, C. D. B. Auditory but not audiovisual cues lead to higher neural sensitivity to the statistical regularities of an unfamiliar musical style. *J. Cogn. Neurosci.* **32**, 2241–2259 (2020). 32762519 10.1162/jocn_a_01614

[39] Bosnyak, D. J., Eaton, R. A. & Roberts, L. E. Distributed auditory cortical representations are modified when non-musicians are trained at pitch discrimination with 40 Hz amplitude modulated tones. *Cereb. Cortex* **14**, 1088–1099 (2004). 15115745 10.1093/cercor/bhh068

[40] Holroyd, C. B. & Coles, M. G. The neural basis of human error processing: Reinforcement learning, dopamine, and the error-related negativity. *Psychol. Rev.* **109**, 679 (2002). 12374324 10.1037/0033-295X.109.4.679

[41] Hoy, C. W., Steiner, S. C. & Knight, R. T. Single-trial modeling separates multiple overlapping prediction errors during reward processing in human EEG. *Commun. Biol.* **4**, 910 (2021). 34302057 10.1038/s42003-021-02426-1 PMC8302587

[42] Luft, C. D. B. Learning from feedback: The neural mechanisms of feedback processing facilitating better performance. *Behav. Brain Res.* **261**, 356–368 (2014). 24406725 10.1016/j.bbr.2013.12.043

[43] Chen, Q. *et al.* Mapping the creative personality: A psychometric network analysis of highly creative artists and scientists. *Creat. Res. J.* **35**, 455–470 (2023). 10.1080/10400419.2023.2184558

[44] Kaufman, S. B. *et al.* Openness to experience and intellect differentially predict creative achievement in the arts and sciences. *J. Personal.* **84**, 248–258 (2016). 10.1111/jopy.12156 PMC4459939 25487993

[45] Silvia, P. J., Nusbaum, E. C., Berg, C., Martin, C. & O’Connor, A. Openness to experience, plasticity, and creativity: Exploring lower-order, high-order, and interactive effects. *J. Res. Personal.* **43**, 1087–1090 (2009). 10.1016/j.jrp.2009.04.015

[46] Zare, M. & Flinchbaugh, C. Voice, creativity, and big five personality traits: A meta-analysis. *Hum. Perform.* **32**, 30–51 (2019). 10.1080/08959285.2018.1550782

[47] Lloyd-Cox, J., Pickering, A. & Bhattacharya, J. Evaluating creativity: How idea context and rater personality affect considerations of novelty and usefulness. *Creat. Res. J.* **34**, 373–390 (2022). 10.1080/10400419.2022.2125721

[48] Runco, M. A. & Jaeger, G. J. The standard definition of creativity. *Creat. Res. J.* **24**, 92–96 (2012). 10.1080/10400419.2012.650092

[49] Müllensiefen, D., Gingras, B., Musil, J. & Stewart, L. The musicality of non-musicians: An index for assessing musical sophistication in the general population. *PLoS One* **9**, e89642 (2014). 24586929 10.1371/journal.pone.0089642 PMC3935919

[50] Rohrmeier, M., Rebuschat, P. & Cross, I. Incidental and online learning of melodic structure. *Conscious. Cogn.* **20**, 214–222 (2011). 20832338 10.1016/j.concog.2010.07.004

[51] Pearce, M. T. The construction and evaluation of statistical models of melodic structure in music perception and composition. (2005).

[52] Pearce, M. T. Statistical learning and probabilistic prediction in music cognition: Mechanisms of stylistic enculturation. *Ann. N.Y. Acad. Sci.* **1423**, 378–395 (2018). 29749625 10.1111/nyas.13654 PMC6849749

[53] Hansen, N. C., Vuust, P. & Pearce, M. ‘ If you have to ask, you’ll never know’: Effects of specialised stylistic expertise on predictive processing of music. *PLoS One* **11**, e0163584 (2016). 27732612 10.1371/journal.pone.0163584 PMC5061385

[54] Hansen, N. C. & Pearce, M. T. Predictive uncertainty in auditory sequence processing. *Front. Psychol.* **5**, 88945 (2014). 10.3389/fpsyg.2014.01052 PMC4171990 25295018

[55] Omigie, D. *et al.* Intracranial recordings and computational modeling of music reveal the time course of prediction error signaling in frontal and temporal cortices. *J. Cogn. Neurosci.* **31**, 855–873 (2019). 30883293 10.1162/jocn_a_01388

[56] Pearce, M. T., Ruiz, M. H., Kapasi, S., Wiggins, G. A. & Bhattacharya, J. Unsupervised statistical learning underpins computational, behavioural, and neural manifestations of musical expectation. *NeuroImage* **50**, 302–313 (2010). 20005297 10.1016/j.neuroimage.2009.12.019

[57] Sears, D. R., Pearce, M. T., Caplin, W. E. & McAdams, S. Simulating melodic and harmonic expectations for tonal cadences using probabilistic models. *J. New Music Res.* **47**, 29–52 (2018). 10.1080/09298215.2017.1367010

[58] Agres, K., Abdallah, S. & Pearce, M. Information-theoretic properties of auditory sequences dynamically influence expectation and memory. *Cogn. Sci.* **42**, 43–76 (2018). 28121017 10.1111/cogs.12477

[59] Brainard, D. H. & Vision, S. The psychophysics toolbox. *Spat. Vis.* **10**, 433–436 (1997). 9176952 10.1163/156856897X00357

[60] Delorme, A. & Makeig, S. EEGLAB: An open source toolbox for analysis of single-trial EEG dynamics including independent component analysis. *J. Neurosci. Methods* **134**, 9–21 (2004). 15102499 10.1016/j.jneumeth.2003.10.009

[61] Oostenveld, R., Fries, P., Maris, E. & Schoffelen, J.-M. FieldTrip: Open source software for advanced analysis of MEG, EEG, and invasive electrophysiological data. *Comput. Intell. Neurosci.* **2011**, 1–9 (2011). 21253357 10.1155/2011/156869 PMC3021840

[62] Hajihosseini, A. & Holroyd, C. B. Frontal midline theta and N 200 amplitude reflect complementary information about expectancy and outcome evaluation. *Psychophysiology* **50**, 550–562 (2013). 23521513 10.1111/psyp.12040

[63] Cheung, V. K. *et al.* Uncertainty and surprise jointly predict musical pleasure and amygdala, hippocampus, and auditory cortex activity. *Curr. Biol.* **29**, 4084–4092 (2019). 31708393 10.1016/j.cub.2019.09.067

[64] Berlyne, D. *Conflict, Arousal, and Curiosity* (1960).

[65] Friston, K. The free-energy principle: A unified brain theory?. *Nat. Rev. Neurosci.* **11**, 127–138 (2010). 20068583 10.1038/nrn2787

[66] Oudeyer, P.-Y., Gottlieb, J. & Lopes, M. Intrinsic motivation, curiosity, and learning: Theory and applications in educational technologies. *Progress Brain Res.* **229**, 257–284 (2016). 10.1016/bs.pbr.2016.05.005 27926442

[67] Belfi, A. M. & Loui, P. Musical anhedonia and rewards of music listening: Current advances and a proposed model. *Ann. N. Y. Acad. Sci.* **1464**, 99–114 (2020). 31549425 10.1111/nyas.14241

[68] Huron, D. B. *Sweet Anticipation: Music and the Psychology of Expectation* (MIT Press, 2006).

[69] Koelsch, S., Vuust, P. & Friston, K. Predictive processes and the peculiar case of music. *Trends Cogn. Sci.* **23**, 63–77 (2019). 30471869 10.1016/j.tics.2018.10.006

[70] Meyer, L. *Emotion and Meaning in Music* (University of Chicago Press, 1956).

[71] Salimpoor, V. N., Zald, D. H., Zatorre, R. J., Dagher, A. & McIntosh, A. R. Predictions and the brain: How musical sounds become rewarding. *Trends Cogn. Sci.* **19**, 86–91 (2015). 25534332 10.1016/j.tics.2014.12.001

[72] Vuust, P., Heggli, O. A., Friston, K. J. & Kringelbach, M. L. Music in the brain. *Nat. Rev. Neurosci.* **23**, 287–305 (2022). 35352057 10.1038/s41583-022-00578-5

[73] Ferreri, L. *et al.* Dopamine modulates the reward experiences elicited by music. *Proc. Natl. Acad. Sci.* **116**, 3793–3798 (2019). 30670642 10.1073/pnas.1811878116 PMC6397525

[74] Sloboda, J. A. Music structure and emotional response: Some empirical findings. *Psychol. Music* **19**, 110–120 (1991). 10.1177/0305735691192002

[75] Mencke, I., Omigie, D., Quiroga-Martinez, D. R. & Brattico, E. Atonal music as a model for investigating exploratory behavior. *Front. Neurosci.* **16**, 793163 (2022). 35812236 10.3389/fnins.2022.793163 PMC9256982

[76] Kathios, N., Sachs, M. E., Zhang, E., Ou, Y. & Loui, P. Generating new musical preferences from multi-level mapping of predictions to reward. *bioRxiv* (2023). 10.1177/09567976231214185 PMC13371869 38019607

[77] Daikoku, T., Yatomi, Y. & Yumoto, M. Statistical learning of music-and language-like sequences and tolerance for spectral shifts. *Neurobiol. Learn. Memory* **118**, 8–19 (2015). 10.1016/j.nlm.2014.11.001 25451311

[78] Omigie, D., Pearce, M. T., Williamson, V. J. & Stewart, L. Electrophysiological correlates of melodic processing in congenital amusia. *Neuropsychologia* **51**, 1749–1762 (2013). 23707539 10.1016/j.neuropsychologia.2013.05.010

[79] Zioga, I., Luft, C. D. B. & Bhattacharya, J. Musical training shapes neural responses to melodic and prosodic expectation. *Brain Res.* **1650**, 267–282 (2016). 27622645 10.1016/j.brainres.2016.09.015 PMC5069926

[80] Mencke, I. *et al.* Prediction under uncertainty: Dissociating sensory from cognitive expectations in highly uncertain musical contexts. *Brain Res.* **1773**, 147664 (2021). 34560052 10.1016/j.brainres.2021.147664

[81] Quiroga-Martinez, D. R. *et al.* Decomposing neural responses to melodic surprise in musicians and non-musicians: Evidence for a hierarchy of predictions in the auditory system. *NeuroImage* **215**, 116816 (2020). 32276064 10.1016/j.neuroimage.2020.116816

[82] Goldman, A., Harrison, P. M., Jackson, T. & Pearce, M. T. Reassessing syntax-related ERP components using popular music chord sequences: A model-based approach. *Music Percept. Interdiscip. J.* **39**, 118–144 (2021). 10.1525/mp.2021.39.2.118

[83] Miranda, R. A. & Ullman, M. T. Double dissociation between rules and memory in music: An event-related potential study. *Neuroimage* **38**, 331–345 (2007). 17855126 10.1016/j.neuroimage.2007.07.034 PMC2186212

[84] Pearce, M. T., Müllensiefen, D. & Wiggins, G. A. The role of expectation and probabilistic learning in auditory boundary perception: A model comparison. *Perception* **39**, 1365–1389 (2010). 21180358 10.1068/p6507

[85] Kern, P., Heilbron, M., de Lange, F. P. & Spaak, E. Cortical activity during naturalistic music listening reflects short-range predictions based on long-term experience. *Elife* **11**, e80935 (2022). 36562532 10.7554/eLife.80935 PMC9836393

[86] Koelsch, S., Gunter, T., Friederici, A. D. & Schröger, E. Brain indices of music processing: ‘nonmusicians’ are musical. *J. Cogn. Neurosci.* **12**, 520–541 (2000). 10931776 10.1162/089892900562183

[87] Koelsch, S., Jentschke, S., Sammler, D. & Mietchen, D. Untangling syntactic and sensory processing: An ERP study of music perception. *Psychophysiology* **44**, 476–490 (2007). 17433099 10.1111/j.1469-8986.2007.00517.x

[88] Auksztulewicz, R. & Friston, K. Repetition suppression and its contextual determinants in predictive coding. *Cortex* **80**, 125–140 (2016). 26861557 10.1016/j.cortex.2015.11.024 PMC5405056

[89] Todorovic, A. & de Lange, F. P. Repetition suppression and expectation suppression are dissociable in time in early auditory evoked fields. *J. Neurosci.* **32**, 13389–13395 (2012). 23015429 10.1523/JNEUROSCI.2227-12.2012 PMC6621367

[90] Przysinda, E., Zeng, T., Maves, K., Arkin, C. & Loui, P. Jazz musicians reveal role of expectancy in human creativity. *Brain Cogn.* **119**, 45–53 (2017). 29028508 10.1016/j.bandc.2017.09.008

[91] Orr, M. G. & Ohlsson, S. Relationship between complexity and liking as a function of expertise. *Music Percept.* **22**, 583–611 (2005). 10.1525/mp.2005.22.4.583

[92] Limb, C. J. & Braun, A. R. Neural substrates of spontaneous musical performance: An fMRI study of jazz improvisation. *PLoS One* **3**, e1679 (2008). 18301756 10.1371/journal.pone.0001679 PMC2244806

[93] Lu, J. *et al.* The brain functional state of music creation: An fMRI Study of Composers. *Sci. Rep.* **5**, 12277 (2015). 26203921 10.1038/srep12277 PMC4512184

[94] Zatorre, R. J. & Salimpoor, V. N. From perception to pleasure: Music and its neural substrates. *Proc. Natl. Acad. Sci.* **110**, 10430–10437 (2013). 23754373 10.1073/pnas.1301228110 PMC3690607

