# Supplementary material for: The association between liking, learning and creativity in music
Source: Sci Rep. 2024 Aug 16;14:19048. doi: 10.1038/s41598-024-70027-z (PMC11329743; doi:10.1038/s41598-024-70027-z)
Supplement: Supplementary file 1 — Supplementary Figure S1. [file 41598_2024_70027_MOESM1_ESM.pdf]

A.

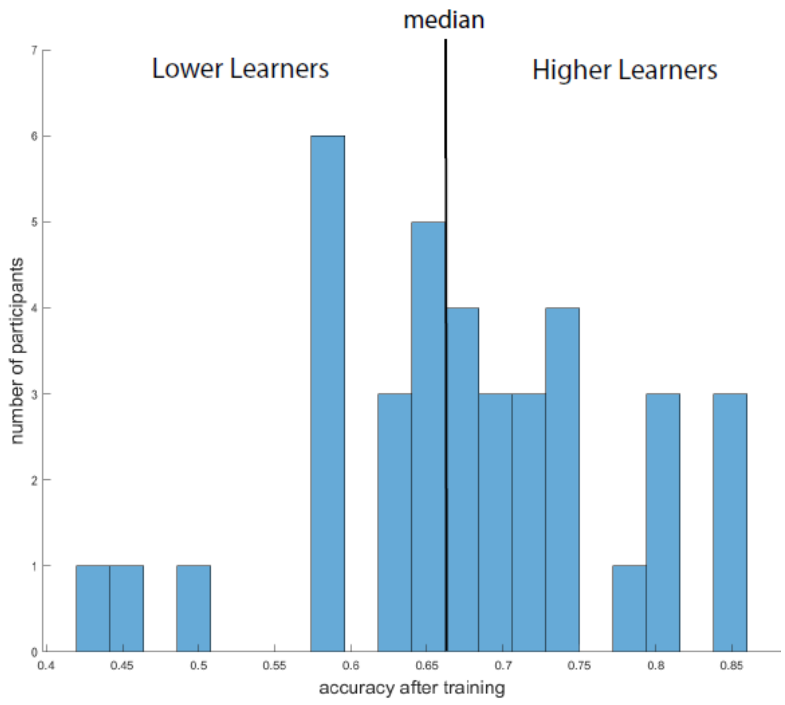

B.

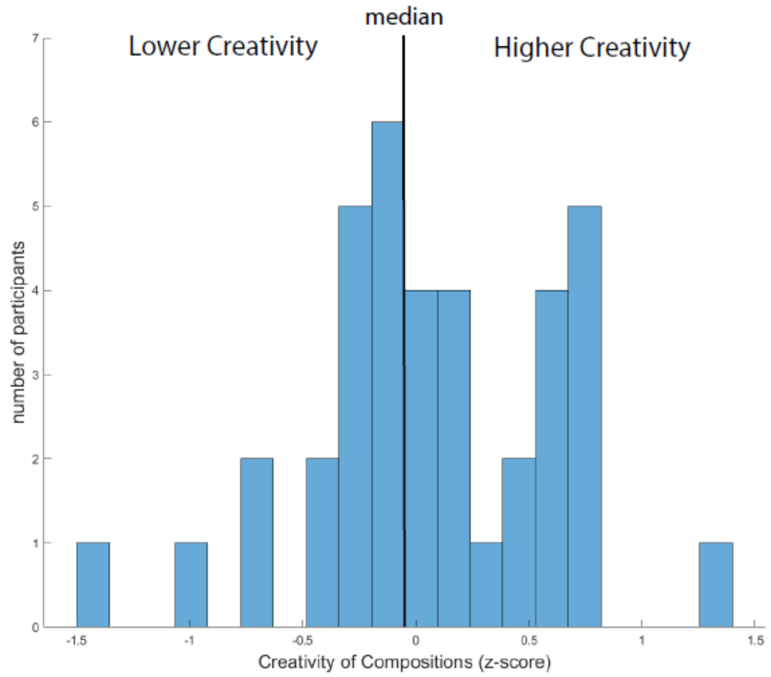

Fig.S1. Histograms showing the data distribution of the following: **A.** The accuracy in the post-test session, calculated as the proportion of correct responses to the test notes after training was finished. The median value used to split the learning groups is represented as a solid vertical line; A Shapiro-wilk test of normality shows that the accuracy data is normally distributed (*p = .537).* **B.** The average creativity ratings of the participants' compositions in the post-test session (i.e. after training). A Shapiro-wilk test of normality shows that the creativity score data is normally distributed (*p = .374).* The median value used to split the creativity groups is marked as the solid vertical line.
